# Construction Notes

**Published:** 1895-11-02

**Authors:** 


### CONSTRUCTION NOTES.

### Bradford Infirmary Extensions.

This extension work, just effected, comprises three observation wards, a room for cases which may come in during the night, additional rooms for nurses (who have latterly been without adequate accommodation), a new room for post-mortem examinations, and a spacious and lofty kitchen fitted with the most modern cooking apparatus, including a patent kitchener by Messrs. Leggott and Marsh, which will, it is calculated, effect a saving to the board of management of £200 a-year as compared with the old system. Messrs. Leggott and Co. have also supplied the Abbotsford stoves, which add to the comfortable appearance of the new buildings. The plans of the new buildings, the estimated cost of which was £6,000, were prepared by Messrs. Milnes and France, of Bradford.

